# Metabolic Energy of Action Potentials Modulated by Spike Frequency Adaptation

**DOI:** 10.3389/fnins.2016.00534

**Published:** 2016-11-17

**Authors:** Guo-Sheng Yi, Jiang Wang, Hui-Yan Li, Xi-Le Wei, Bin Deng

**Affiliations:** ^1^School of Electrical Engineering and Automation, Tianjin UniversityTianjin, China; ^2^School of Automation and Electrical Engineering, Tianjin University of Technology and EducationTianjin, China

**Keywords:** spike frequency adaptation, *I*_M_, *I*_AHP_, metabolic energy, action potential, conductance-based model

## Abstract

Spike frequency adaptation (SFA) exists in many types of neurons, which has been demonstrated to improve their abilities to process incoming information by synapses. The major carrier used by a neuron to convey synaptic signals is the sequences of action potentials (APs), which have to consume substantial metabolic energies to initiate and propagate. Here we use conductance-based models to investigate how SFA modulates the AP-related energy of neurons. The SFA is attributed to either calcium-activated K^+^ (*I*_AHP_) or voltage-activated K^+^ (*I*_M_) current. We observe that the activation of *I*_AHP_ or *I*_M_ increases the Na^+^ load used for depolarizing membrane, while produces few effects on the falling phase of AP. Then, the metabolic energy involved in Na^+^ current significantly increases from one AP to the next, while for K^+^ current it is less affected. As a consequence, the total energy cost by each AP gets larger as firing rate decays down. It is also shown that the minimum Na^+^ charge needed for the depolarization of each AP is unaffected during the course of SFA. This indicates that the activation of either adaptation current makes APs become less efficient to use Na^+^ influx for their depolarization. Further, our simulations demonstrate that the different biophysical properties of *I*_M_ and *I*_AHP_ result in distinct modulations of metabolic energy usage for APs. These investigations provide a fundamental link between adaptation currents and neuronal energetics, which could facilitate to interpret how SFA participates in neuronal information processing.

## Introduction

Neurons in the central nervous system (CNS) have powerful ability to encode and conduct afferent information, which requires enormous amounts of metabolic energy to realize this function (Attwell and Laughlin, 2001; Alle et al., 2009; Harris et al., 2012; Bowie and Attwell, 2015). They use sequences of action potentials (APs) as the principal carrier to accurately convey synaptic signals to the target cells (Koch, 1999; Kandel et al., 2012). The generation and conduction of APs arises from the flow of ions, such as Na^+^ or K^+^, through their voltage-gated channels, which accounts for a large proportion of overall energy usage by neurons (Attwell and Laughlin, 2001; Alle et al., 2009; Sengupta et al., 2010; Moujahid et al., 2011, 2014; Harris et al., 2012; Kandel et al., 2012; Moujahid and d'Anjou, 2012; Bowie and Attwell, 2015). In particular, the concentration gradients of ions during an AP have to be restored against their electrochemical gradients by associated pumps, which consumes substantial energies provided by the hydrolysis of adenosine triphosphate (ATP) molecules (Sengupta et al., 2010; Moujahid et al., 2011, 2014; Harris et al., 2012; Kandel et al., 2012; Moujahid and d'Anjou, 2012). The energy cost of APs is tightly related to neural information processing and computational function (Laughlin, 2001; Sengupta et al., 2010, 2013, 2014; Harris et al., 2012; Kann et al., 2014; Bowie and Attwell, 2015). Using neuron models to extrapolate the energetics involved in different patterns of AP trains is therefore necessary for capturing the full strategies adopted by a neuron to encode information.

To effectively encode synaptic signals, there are various patterns of spike trains generated by CNS neurons. One special pattern is that the onset firing rate of the cell is high and then decays down to a lower steady-state level during prolonged constant stimulus, which is referred to as spike-frequency adaptation (SFA) (Wang, 1998; Wark et al., 2007; Prescott and Sejnowski, 2008; Benda et al., 2010). This is a common firing pattern exhibited by many types of neurons, which has been shown to effectively shape their signal processing properties on timescales larger than tens of ms (Pineda et al., 1999; Sharpee et al., 2006; Wark et al., 2007; Prescott and Sejnowski, 2008; Benda et al., 2010). Within the mechanisms that may lead to SFA, the slow adaptation currents are of particular importance. Two primary types of such current include M-type current (*I*_M_) (Brown and Adams, 1980) and AHP-type current (*I*_AHP_) (Madison and Nicoll, 1984). *I*_M_ is a slow, voltage-gated K^+^ current, which is activated prior to spike initiation. *I*_AHP_ is a calcium-gated K^+^ current, and its activation is spike-dependent, which cannot occur at the subthreshold voltages. Both of two inhibitory K^+^ currents are able to participate in AP initiation and reduce firing rate once activated. In particular, the disparate activation basis for *I*_M_ and *I*_AHP_ endows them with distinct modulatory effects on neural coding (Ermentrout, 1998; Wang, 1998; Liu and Wang, 2001; Benda and Herz, 2003; Prescott et al., 2006; Prescott and Sejnowski, 2008; Ladenbauer et al., 2014; Yi et al., 2015b). However, it has attracted little attention about how two adaptation currents impact the energetics of a neuron. Addressing it could provide deep insights into how *I*_M_ or *I*_AHP_ participates in AP generation, which is also an essential step toward interpreting the mechanism underlying their modulations of neural information processing.

In fact, exploring neural computation from the point of view of energy metabolism has always been the issue of concern in neuroscience. It has made major progress with regard to information processing (Kann et al., 2014; Sengupta et al., 2014; Bowie and Attwell, 2015), energy utilization (Howarth et al., 2012), development (Blomgren et al., 2003; Schuchmann et al., 2005) and survival (Diaz et al., 2012) of neurons. The energy “budget” for neural signaling indicates that the APs make a significant contribution to the overall usage (Howarth et al., 2012). One common method used for estimating the AP-related energy is to calculate the amount of Na^+^ or K^+^ involved in it, which is also known as ion counting approach (Attwell and Laughlin, 2001; Alle et al., 2009; Sengupta et al., 2010, 2013, 2014). It is performed by integrating the area of ionic current underlying the AP being considered, which is also defined as the Na^+^ or K^+^ load of the AP. It represents the total amount of ion used to depolarize or hyperpolarize membrane during the AP. The Na^+^-K^+^ pump hydrolyses one ATP to adenosine diphosphate (ADP) when it exports three Na^+^ ions out of the cell or import two K^+^ ions into the cell (Kandel et al., 2012). Then, one can estimate the number of ATP molecules hydrolyzed by Na^+^ or K^+^ pump according to their ion counting. With this method, the effects of cell size, AP shape (i.e., height and width), channel density, and kinetics, graded potentials, temperature, and stimulus statistics on the energy cost of APs have been investigated (Alle et al., 2009; Sengupta et al., 2010, 2013, 2014). They find that the energy consumption of a neuron is strongly dependent on its firing rate or interspike interval (ISI), which could detect the transition of the cell from quiescence to firing state. It is also shown that the AP is very inefficient since there is temporal overlap between inward Na^+^ and outward K^+^ currents (Hodgkin, 1975; Crotty et al., 2006; Alle et al., 2009; Carter and Bean, 2009). Such overlap leads the total Na^+^ load to exceed the minimum load that is required for the depolarization of AP, and then makes it inefficient. This is a major determinant of energy efficiency, and varies greatly between cell types. For example, the squid giant axons are highly inefficient, whereas the thalamo-cortical neurons in rats are highly efficient (Hodgkin, 1975; Sengupta et al., 2010; Howarth et al., 2012). Due to the temporal overlaps, the ion counting approach may introduce obvious uncertainties in measuring the number of Na^+^ influx, which results in the overestimation of energy values (Attwell and Laughlin, 2001; Alle et al., 2009; Sengupta et al., 2010; Moujahid et al., 2011, 2014; Howarth et al., 2012; Moujahid and d'Anjou, 2012).

Recently, Moujahid et al. (2011), Moujahid et al. (2014), Moujahid and d'Anjou (2012) propose an alternative way for estimating the energy cost of APs. Unlike ion counting approach, their energy estimation does not require the stoichiometry of Na^+^ or K^+^. It is based on the biophysical nature of conductance-based neuron models, which enables one to deduce an analytical expression of the metabolic energy involved in the resting or firing states of the cell. Since there is no hypothesis about the temporal overlaps between Na^+^ and K^+^ currents, it avoids the overestimation of energy induced by ion counting. More importantly, it allows one to quantify the metabolic energy involved in each ionic channel, which offers the possibility to determine their contributions to the total energy cost associated with neural computation. With this approach, Moujahid et al have successfully identified the energy efficiency involved in the spiking cells from neocortex, hippocampus, thalamus, and squid axon (Moujahid et al., 2011, 2014; Moujahid and d'Anjou, 2012). They have also elaborated the relationships between firing rate, temperature, information transmission, and the metabolic energy they consume. Based on their method, Ju et al. (2016) have estimated the energy consumption by AP conduction along axons with different geometric shapes; Li et al. (2016) have investigated the energy cost of the seizure-like discharges induced by abnormal astrocytic glutamate oscillation; our earlier study (Yi et al., 2015a) has characterized the energy efficiency of neurons associated with different dynamics of firing threshold. These earlier investigations attest the predictive power of this method for quantifying the metabolic energy involved in the dynamics of biophysical model neurons.

Here we set out to identify how adaptation currents modulate the energy cost of a neuron as they reduce firing rate. To achieve this goal, the conductance-based models that involve two ionic mechanisms of SFA, i.e., *I*_M_ and *I*_AHP_, are introduced in present study. The energy cost related to the simulated APs is determined by the method proposed by Moujahid et al. (2011). With this approach, we have characterized in detail the AP-related energy and AP efficiency during the course of SFA. Meanwhile, the impacts of introduced adaptation mechanisms on the energies consumed by each ionic current underlying relevant AP are also identified.

## Model and methods

### Neuron model with adaptation

**Figure 1:**
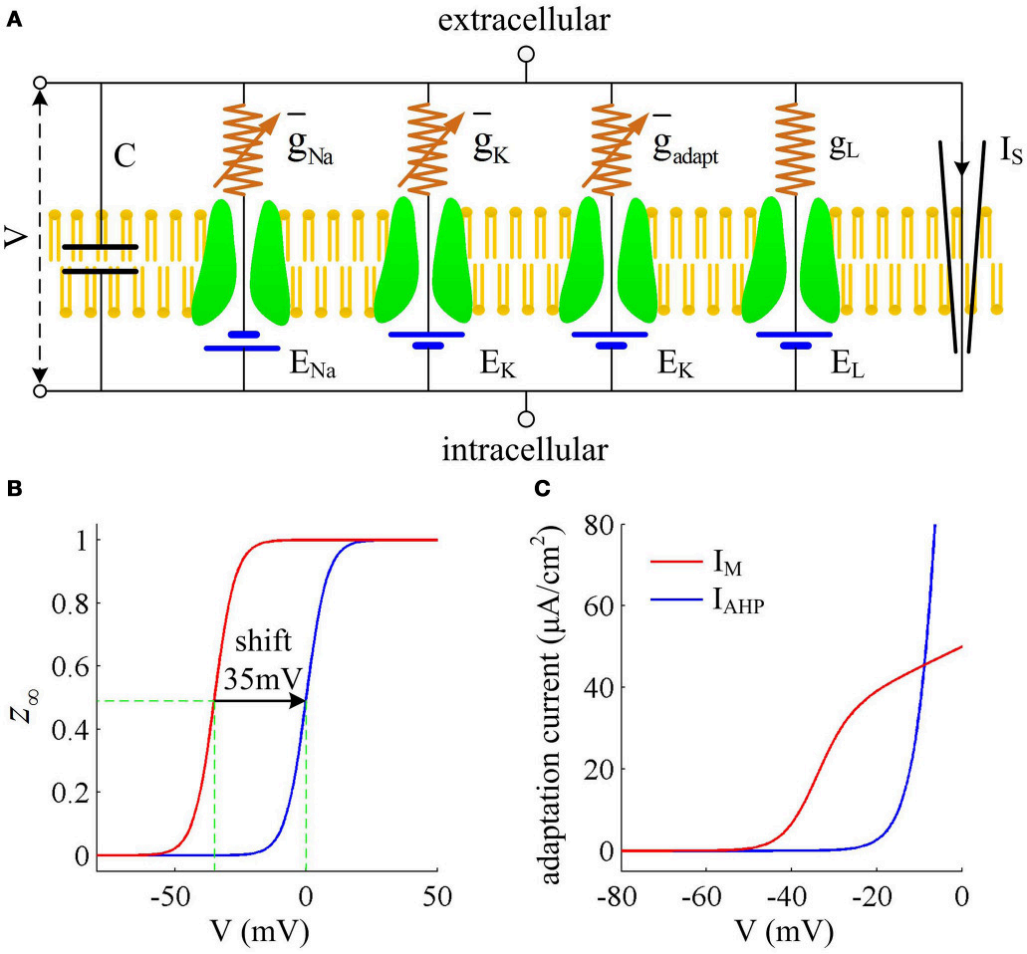
**Prescott model and the activation of adaptation currents. (A)** Schematic of ionic currents and conductances for Prescott model. **(B)** Steady-state activation function *z*_∞_(*V*) for *I*_M_ and *I*_AHP_ currents. **(C)** Relationship between steady-state adaptation current and membrane potential *V*.

We consider a conductance-based neuron model in our simulations, which is the Prescott model, as shown in Figure 1A. It involves four ionic channels on its cell membrane, which are fast Na^+^ current (*I*_Na_), delayed rectifying K^+^ current (*I*_K_), slowly activated adaptation current (*I*_adapt_), and passive leak current (*I*_L_), respectively. This model is modified from original Morris-Lecar (ML) model by Prescott and Sejnowski (2008) to investigate how SFA participates in neural coding. The inward *I*_Na_ and outward *I*_K_ are two essential ions for generating APs. The addition of adaptation current *I*_adapt_ is used for modulating spike initiation dynamics and generating SFA. The differential equations describing the dynamics of the Prescott model are (Prescott and Sejnowski, 2008)

(1) $$\begin{array}{lll} \text{C}\frac{\text{d}V}{\text{d}t} & = & I_{\text{S}}-I_{\text{Na}}-I_{\text{K}}-I_{\text{adapt}}-I_{\text{L}} \end{array}$$

(2) $$\begin{array}{lll} \frac{\text{d}n}{\text{d}t} & = & \varphi \frac{n_{\infty }\left(V\right)-n}{\tau_{n}\left(V\right)} \end{array}$$

(3) $$\begin{array}{lll} \frac{\text{d}z}{\text{d}t} & = & \frac{z_{\infty }\left(V\right)-z}{\tau_{z}} \end{array}$$

where *V* is the membrane potential, and *n, z* are the activation variables for K^+^ and adaptation currents. C is the membrane capacitance and φ is the scale parameter for variable *n*. *I*_S_ is the applied current, and the ionic currents used in above are

(4) $$\begin{array}{lll} I_{\text{Na}} & = & {\bar{\text{g}}}_{\text{Na}}m_{\infty }\left(V\right)\left(V-{\text{E}}_{\text{Na}}\right) \end{array}$$

(5) $$\begin{array}{lll} I_{\text{K}} & = & {\bar{\text{g}}}_{\text{K}}n\left(V-{\text{E}}_{\text{K}}\right) \end{array}$$

(6) $$\begin{array}{lll} I_{\text{adapt}} & = & {\bar{\text{g}}}_{\text{adapt}}z\left(V-{\text{E}}_{\text{K}}\right) \end{array}$$

(7) $$\begin{array}{lll} I_{\text{L}} & = & {\text{g}}_{\text{L}}\left(V-{\text{E}}_{\text{L}}\right) \end{array}$$

Here ${\bar{\text{g}}}_{\text{Na}}$, ${\bar{\text{g}}}_{\text{K}}$, ${\bar{\text{g}}}_{\text{adapt}}$, g_L_ are the maximum conductances associated with relevant ionic currents, and E_Na_, E_K_, E_L_ are their reversal potentials. The steady-state activation and time functions are

(8) $$\begin{array}{l} m_{\infty }(V)=0.5\left[1+\tanh\left(\frac{V-{\text{B}}_{m}}{{\text{A}}_{m}}\right)\right]\\ n_{\infty }(V)=0.5\left[1+\tanh\left(\frac{V-{\text{B}}_{n}}{{\text{A}}_{n}}\right)\right]\\ z_{\infty }(V)=1/\left[1+\exp\left(\frac{{\text{B}}_{z}-V}{{\text{A}}_{z}}\right)\right]\\ \tau_{n}(V)=1/\cosh\left(\frac{V-{\text{B}}_{n}}{2{\text{A}}_{n}}\right) \end{array}$$

**Figure 2:**
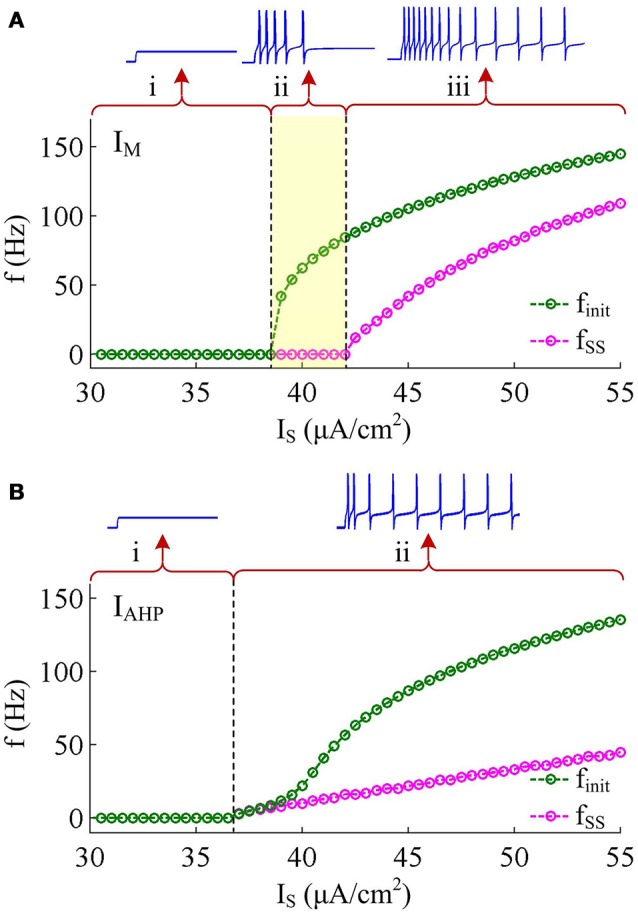
**Response properties of the Prescott neuron with *I*_M_ or *I*_AHP_. (A)** Firing rate as a function of constant current *I*_S_ (i.e., *f*_init_ − *I*_S_ and *f*_SS_ − *I*_S_ curves) in the case of *I*_M_ -mediated adaptation. There are three regions, which are (i) subthreshold oscillation, (ii) no repetitive firing after adaptation, and (iii) repetitive firing with low rate after adaptation. **(B)** *f*_init_ − *I*_S_ and *f*_SS_ − *I*_S_ curves in the case of *I*_AHP_ -mediated adaptation. There are only two regions, which are (i) subthreshold oscillation and (ii) repetitive firing with low rate after adaptation.

Two separate adaptation currents are examined in our simulations, which are *I*_M_ and *I*_AHP_. For the model with *I*_M_ current, we set ${\bar{\text{g}}}_{\text{adapt}}={\bar{\text{g}}}_{\text{M}}=0.5\text{mS}/\text{c}{\text{m}}^{2}$, B_*z*_ = −35mV, A_*z*_ = 4mV and τ_*z*_ = 100ms (Prescott et al., 2006; Prescott and Sejnowski, 2008). For the model with *I*_AHP_ current, the relevant parameters are ${\bar{\text{g}}}_{\text{adapt}}={\bar{\text{g}}}_{\text{AHP}}=5\text{mS}/\text{c}{\text{m}}^{2}$, B_*z*_ = 0mV, A_*z*_ = 4mV and τ_*z*_ = 100ms (Prescott et al., 2006; Prescott and Sejnowski, 2008). As mentioned in Introduction, the critical distinction between *I*_M_ and *I*_AHP_ is their activation voltage, which is governed by parameter B_*z*_. As shown in Figures 1B,C, the half-activation voltage for *I*_M_ is −35mV, which allows it to activate at subthreshold potentials, i.e., spike independent. However, the half-activation voltage for *I*_AHP_ is 0 mV, which is a depolarized value above the threshold voltage for AP initiation. Then, *I*_AHP_ current only activates during APs, i.e., spike dependent. Such different activations of *I*_M_ and *I*_AHP_ have been shown to result in distinct modulations of spike trains (Prescott et al., 2006; Prescott and Sejnowski, 2008; Yi et al., 2015b). Specifically, *I*_M_ is able to become sufficiently strong to stabilize *V* at a subthreshold voltage and then terminates repetitive spiking elicited by constant current *I*_S_ (Figure 2A), whereas *I*_AHP_ only reduces firing rate (Figure 2B). In our simulations, we only consider the case that one form of adaptation is present, i.e., either *I*_M_ (${\bar{\text{g}}}_{\text{AHP}}=0\text{mS}/\text{c}{\text{m}}^{2}$) or *I*_AHP_ (${\bar{\text{g}}}_{\text{M}}=0\text{mS}/\text{c}{\text{m}}^{2}$).

Other parameters in Equations (1)–(8) are as follows: C = 2μF/cm^2^, E_Na_ = 50mV, E_K_ = −100mV, E_L_ = −70mV, ${\bar{\text{g}}}_{\text{Na}}=20\text{mS}/\text{c}{\text{m}}^{2}$, ${\bar{\text{g}}}_{\text{K}}=20\text{mS}/\text{c}{\text{m}}^{2}$, ${\text{g}}_{\text{L}}=2\text{mS}/\text{c}{\text{m}}^{2}$, B_*m*_ = −1.2mV, A_*m*_ = 18mV, B_*n*_ = 0mV, A_*n*_ = 10mV, and φ = 0.15. Note that the biophysical parameters used in Prescott model are the same as those described in Prescott and Sejnowski (2008), which are determined by systematically varying them to reproduce the experimentally observed SFA in hippocampal CA1 pyramidal neurons.

### Energy cost of neuron model

The metabolic energy involved in the conductance-based models is determined by the novel approach proposed by Moujahid et al. (2011), which is not based on the stoichiometry of Na^+^ ions and then avoids the overestimation of energy. Here, we regard the Prescott model shown in Figure 1A as an electrical circuit that is consisted of membrane capacitance C, Na^+^, K^+^, adaptation, and leak channels. The batteries in this circuit are the reversal potential of each ionic channel. Following the descriptions by Moujahid et al. (2011), the total electrical energy involved in this neural circuit at a given time can be written as

(9) $$\begin{array}{l} D\left(t\right)=0.5\text{C}V^{2}+D_{\text{Na}}+D_{\text{K}}+D_{\text{adapt}}+D_{\text{L}} \end{array}$$

where 0.5C*V*^2^ on the right side stands for the electrical energy accumulated in membrane capacitance. The other four terms, i.e., *D*_Na_, *D*_K_, *D*_adapt_, *D*_L_, are the respective energy associated with each battery, which are needed to create the concentration jump for corresponding ion. It is known that the rate of electrical energy provided to the circuit by a battery is its electromotive force multiplied by the current flowing through the battery. Then, the first-order derivative of Equation (9) with respect to time *t* can be expressed by

(10) $$\begin{array}{l} \frac{\text{d}D}{\text{d}t}=\text{C}V\frac{\text{d}V}{\text{d}t}+I_{\text{Na}}{\text{E}}_{\text{Na}}+I_{\text{K}}{\text{E}}_{\text{K}}+I_{\text{adapt}}{\text{E}}_{\text{adapt}}+I_{\text{L}}{\text{E}}_{\text{L}} \end{array}$$

where E_adapt_ = E_K_. In the following, this energy per second is denoted by letter δ. If we substitute $\text{C}\frac{\text{d}V}{\text{d}t}$ with Equation (1), the above energy rate δ of the circuit becomes

(11) $$\begin{array}{lll} \delta & = & I_{\text{S}}V-I_{\text{Na}}\left(V-{\text{E}}_{\text{Na}}\right)-I_{\text{K}}\left(V-{\text{E}}_{\text{K}}\right)-I_{\text{adapt}}\left(V-{\text{E}}_{\text{K}}\right)\\ -I_{\text{L}}\left(V-{\text{E}}_{\text{L}}\right) \end{array}$$

where the first term on the right side is the energy power supplied by applied current *I*_S_, and the last four terms are the energy consumption rate associated with each ionic channel. Substituting *I*_Na_, *I*_K_, *I*_adapt_, *I*_L_ with their expressions, i.e., Equations (4)–(7), we have

(12) $$\begin{array}{lll} \delta & = & I_{\text{S}}V-{\bar{\text{g}}}_{\text{Na}}m_{\infty }\left(V\right){\left(V-{\text{E}}_{\text{Na}}\right)}^{2}-{\bar{\text{g}}}_{\text{K}}n{\left(V-{\text{E}}_{\text{K}}\right)}^{2}\\ -{\bar{\text{g}}}_{\text{adapt}}z{\left(V-{\text{E}}_{\text{K}}\right)}^{2}-{\text{g}}_{\text{L}}{\left(V-{\text{E}}_{\text{L}}\right)}^{2} \end{array}$$

which shows the derivative of the total metabolic energy in Prescott model as a function of its state variable *V, n*, and *z*. From Equation (12), the energy rate for each ionic channel can be given as

(13) $$\begin{array}{lll} \delta_{\text{Na}} & = & {\bar{\text{g}}}_{\text{Na}}m_{\infty }\left(V\right){\left(V-{\text{E}}_{\text{Na}}\right)}^{2} \end{array}$$

(14) $$\begin{array}{lll} \delta_{\text{K}} & = & {\bar{\text{g}}}_{\text{K}}n{\left(V-{\text{E}}_{\text{K}}\right)}^{2} \end{array}$$

(15) $$\begin{array}{lll} \delta_{\text{adapt}} & = & {\bar{\text{g}}}_{\text{adapt}}z{\left(V-{\text{E}}_{\text{K}}\right)}^{2} \end{array}$$

(16) $$\begin{array}{lll} \delta_{\text{L}} & = & {\text{g}}_{\text{L}}{\left(V-{\text{E}}_{\text{L}}\right)}^{2} \end{array}$$

Then, the total energy consumed by a specific AP or its associated ionic currents can be determined by integrating above instantaneous consumption (i.e., δ, δ_Na_, δ_K_, δ_adapt_ or δ_L_) during the AP.

## Results

### Effects of *I*_M_ on the energy consumption of AP

**Figure 3:**
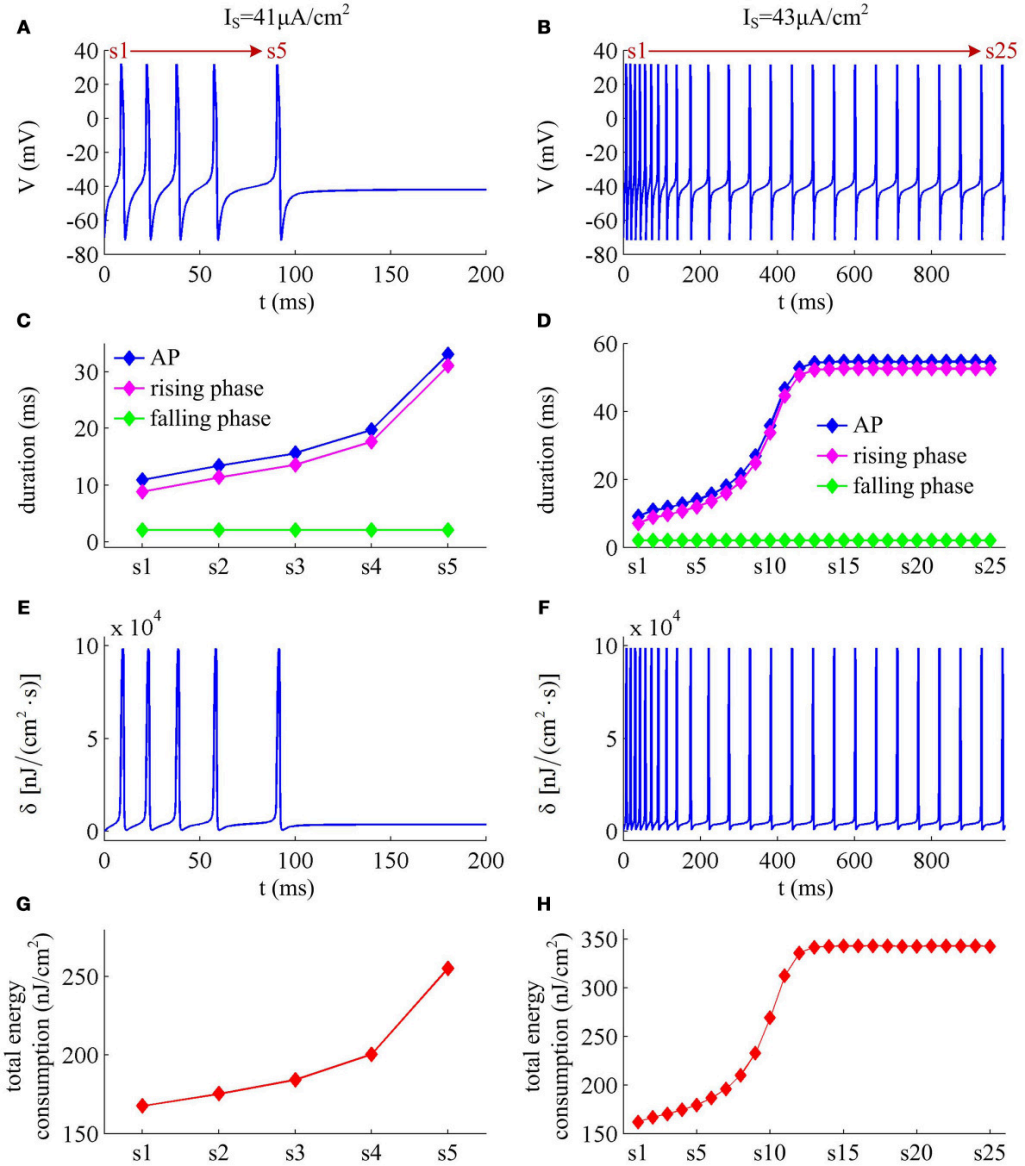
**Energy cost of spike trains generated in Prescott neuron with *I*_M_. (A,B)** respectively show the time course of membrane potential triggered by $I_{\text{S}}=41\mu \text{A}/\text{c}{\text{m}}^{2}$ and 43μA/cm^2^. There are 5 APs initiated with $I_{\text{S}}=41\mu \text{A}/\text{c}{\text{m}}^{2}$, and we number them from s1 to s5. With $I_{\text{S}}=43\mu \text{A}/\text{c}{\text{m}}^{2}$, there are 25 APs in total, which are numbered from s1 to s25. The duration of relevant AP during the course of SFA is depicted in **(C,D)**, which also show their rising and falling phase duration. **(E,F)** are the relevant energy consumption rate of two spike trains. **(G,H)** respectively summarize the energy cost by each AP with two values of *I*_S_.

*I*_M_ -mediated adaptation is able to both terminate repetitive spiking and reduce firing rate. With $I_{\text{S}}=41\mu \text{A}/\text{c}{\text{m}}^{2}$, the Prescott model generates several initial APs to constant current stimulus, and the initial firing rate *f*_init_ (calculated as the reciprocal of the first ISI) at this value of *I*_S_ can reach 113.4 Hz (Figure 3A). As *I*_M_ current activates, the rate of membrane depolarization preceding the spike gets slower and the duration of rising phase gets longer, whereas the duration of the falling phase of the AP is not affected (Figure 3C). As a result, the ISI is extended and the firing rate is reduced during the course of SFA. In steady state, repetitive spiking stops and the firing rate of the neuron becomes 0 Hz, i.e., *f*_SS_ = 0Hz. With higher stimulus (such as, $I_{\text{S}}=43\mu \text{A}/\text{c}{\text{m}}^{2}$), the Prescott neuron continues to spike repetitively at a low rate (*f*_SS_ = 18.3Hz) in its steady state, and the activation of *I*_M_ current only reduces firing rate, as shown in Figures 3B,D.

We respectively characterize the metabolic energies consumed by the simulated APs in above two cases. Figures 3E,F show the instantaneous total energy consumption δ by four ionic channels. One can clearly observe that the total consumptions of metabolic energy during the upstroke and downstroke of AP are much higher than those consumed by subthreshold behaviors, which can reach a peak of near 100000nJ/(cm^2^·s). This is mainly because there are vast ions flowing into and out of the cell during the suprathreshold course of AP, which consumes plenty of metabolic energies. In order to maintain the activities of various ions, such high energy consumption must be supplied by the hydrolysis of ATP molecules to ADP or be replenished by ion pumps (Harris et al., 2012; Kandel et al., 2012; Moujahid and d'Anjou, 2012; Moujahid et al., 2014).

Further, we derive the energy consumption by each AP with two values of *I*_S_. It is found that the total energy consumed by each AP increases as the activation of *I*_M_ reduces firing rate (Figures 3G,H). That is, the energy cost of the AP is an increasing function of its duration during the course of SFA. Once *I*_M_ sufficiently activates, the neuron achieves steady state. In this case, the ISI and firing rate both remains unchanged. Accordingly, the metabolic energy consumed by relevant AP also reaches a steady state, which does not increase with time *t* anymore.

### Effects of *I*_M_ on the energies consumed by ionic currents

**Figure 4:**
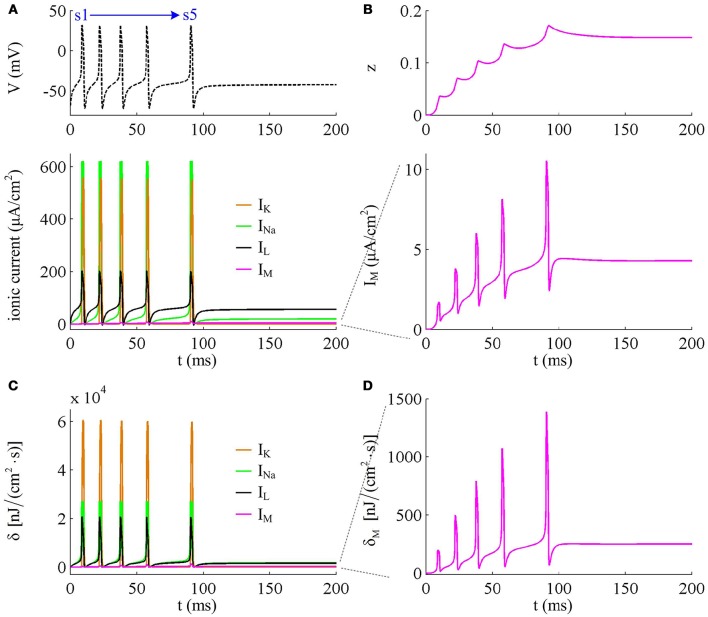
**Energy consumption rate of ionic currents with $I_{\text{S}}=41\mu \text{A}/\text{c}{\text{m}}^{2}$. (A)** *I*_Na_, *I*_K_, *I*_L_, and *I*_M_ underlying the spike trains triggered by $I_{\text{S}}=41\mu \text{A}/\text{c}{\text{m}}^{2}$. Inward Na^+^ current is negative, but we plot it with a positive sign here. **(B)** *I*_M_ current and its activation variable *z*. **(C)** Energy consumption rate δ of each current. **(D)** Closer view of the energy consumption rate of *I*_M_ current (i.e., δ_M_).

**Figure 5:**
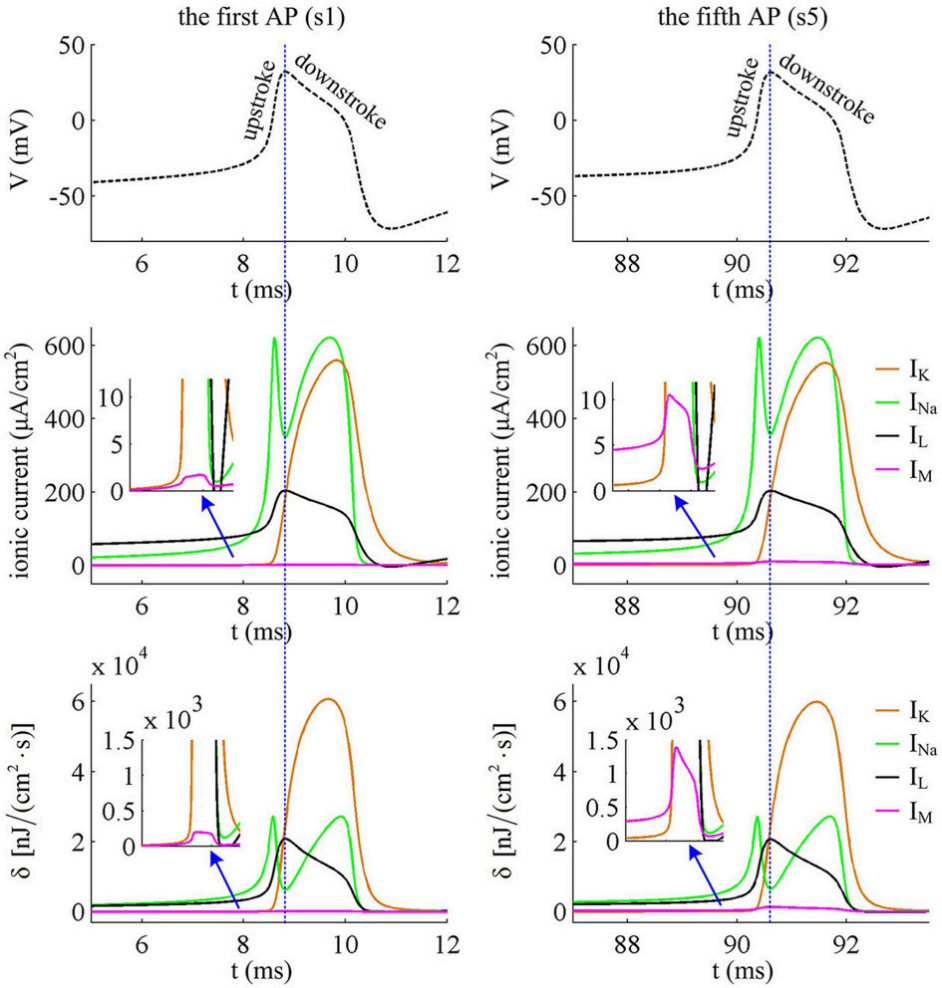
**Closer views of ionic currents and their relevant δ**. The APs are the first and fifth one elicited by $I_{\text{S}}=41\mu \text{A}/\text{c}{\text{m}}^{2}$ in the Prescott neuron with *I*_M_ current.

To analyze in detail how *I*_M_ -mediated adaptation increases the energy consumption of APs, we use Prescott model to characterize the metabolic energy involved in each ionic channel for above two cases. Figures 4A,B respectively show the evolutions of *I*_Na_, *I*_K_, *I*_L_, and *I*_M_ underlying the spike train triggered by $I_{\text{S}}=41\mu \text{A}/\text{c}{\text{m}}^{2}$. Figure 5 is the closer view of ionic currents underlying the first and fifth APs shown in Figure 4A. These ions may flow into or out of the cell according to their ionic concentrations on the inside and the outside of cell membrane. The ionic currents with opposite directions play distinct roles in the generation of AP (Koch, 1999; Kandel et al., 2012; Yi et al., 2014, 2015a,b), and two common ions are Na^+^ and K^+^. Inward *I*_Na_ mainly depolarizes membrane potential *V*, which has to activate earlier during an AP and then drives *V* to produce the fast upstroke (Figure 5). On the contrary, the outward K^+^ current mainly hyperpolarizes membrane potential, which activates after inward Na^+^ and mainly appears in the repolarization period of AP.

In Prescott neuron, the intensity of *I*_Na_ or *I*_K_ underlying an AP is much higher than the other two currents (Figures 4, 5). Especially for inward Na^+^ current, its intensity can reach a peak of about 620μA/cm^2^ during an AP. As a result, the energy consumptions by two active channels are both much higher than that of either *I*_L_ or *I*_M_ (Figures 4C,D). Unlike *I*_Na_ and *I*_K_, the outward leak current *I*_L_ is passive, which does not include gating variables. This makes its intensity and energy cost only vary with membrane potential *V*.

**Figure 6:**
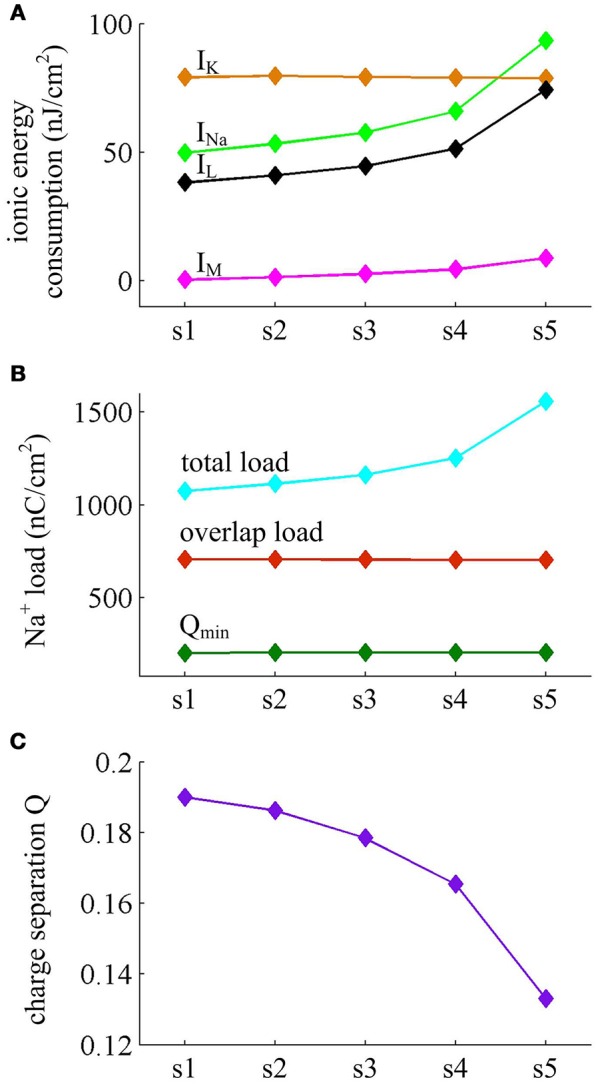
**Energy consumed by each current and Na^+^ load with $I_{\text{S}}=41\mu \text{A}/\text{c}{\text{m}}^{2}$**. **(A)** Energy cost by *I*_Na_, *I*_K_, *I*_L_, and *I*_M_ during each AP with $I_{\text{S}}=41\mu \text{A}/\text{c}{\text{m}}^{2}$. **(B)** Total Na^+^ load, overlap Na^+^ load, and minimum Na^+^ charge Q_min_ during each AP. **(C)** Charge separation Q per spike during the course of SFA.

Compared with Na^+^, K^+^, and leak currents, the intensity of outward *I*_M_ in Prescott neuron is much lower during an AP, which can only increase to a peak of near 12μA/cm^2^ (bottom panel, Figure 4B). Then, the energy consumption of *I*_M_ is much smaller than those consumed by Na^+^, K^+^ or leak channels (Figures 4C, 5). But such weak inhibitory current is able to participate in AP initiation. To be specific, the activation of *I*_M_ antagonizes inward Na^+^ at the subthreshold potentials and decreases the rate of membrane depolarization preceding the spike, which further extends the area of relevant membrane potential. In this case, the neuron has to import more Na^+^ to depolarize its membrane and initiate AP. Then, the total Na^+^ load during an AP increases with the activation of *I*_M_ current (Figure 6B), and accordingly the energy consumption in Na^+^ channel increases from one spike to the next (Figure 6A). Once AP is initiated, the *I*_M_ current produces few effects on suprathreshold firing behaviors, especially during the falling phase of AP (Figures 3C,D). As a result, the energy consumption of outward K^+^ current during one AP is almost unaffected by the activation of *I*_M_ (Figure 6A).

It is worth noting that Na^+^ current has a negative sign because it flows into the cell. But it is plotted with a positive sign here for a better visualization of the temporal overlap between Na^+^ and K^+^ currents during each AP. From Figure 5, one can clearly observe that there are extensive overlaps between inward Na^+^ and outward K^+^ currents, especially for the repolarizing component of AP. The degree of overlap Na^+^ load could be measured as the difference between the total Na^+^ load and the Na^+^ load of depolarizing component (Crotty et al., 2006; Alle et al., 2009; Sengupta et al., 2010; Howarth et al., 2012; Moujahid and d'Anjou, 2012; Moujahid et al., 2014). From Figure 6B, it can be found that the overlap Na^+^ load remains almost unchanged as the activation of *I*_M_ reduces firing rate. As mentioned in Introduction, such overlap makes APs inefficient, and an efficient AP has little overlap. To quantify AP efficiency during the course of SFA, we introduce a dimensionless measure, which is charge separation Q (Alle et al., 2009; Moujahid and d'Anjou, 2012; Moujahid et al., 2014). It is calculated by the ratio of the minimum Na^+^ charge (Q_min_) required for the depolarization of AP and the total Na^+^ charge (Q_Na_) during the AP, i.e., Q = Q_min_/Q_Na_ (Moujahid and d'Anjou, 2012; Moujahid et al., 2014). This measure allows us to quantify how efficiently Na^+^ influx is used for AP depolarization during the course of SFA. According to the description by Carter and Bean (2009), we calculate the minimum charge as Q_min_ = CΔ*V*, where C is the membrane capacitance and Δ*V* is the change in voltage during the AP. It refers to the Na^+^ charge per spike that is not counterbalanced by any K^+^ current, i.e., the K^+^ repolarization current *I*_K_ and adaptation current *I*_M_. During the course of SFA, the minimum charge Q_min_ calculated in this way remains unchanged from one AP to the next (Figure 6B). However, the charge separation Q of each AP is reduced as *I*_M_ extends its duration (Figure 6C). This indicates that the activation of *I*_M_ makes the AP in Prescott neuron get less efficient to use Na^+^ influx to generate its depolarization. Here the first AP is the most efficient with a charge separation approaching 19%, while the fifth AP is the most inefficient with only 13.2% of Na^+^ entry used for AP depolarization. As the activation of *I*_M_ reduces firing rate, the Na^+^ influx becomes less efficient in inducing the depolarization of AP since it has to compete with this adaptation current, which is just like it does during the overlap with the repolarizing *I*_K_.

**Figure 7:**
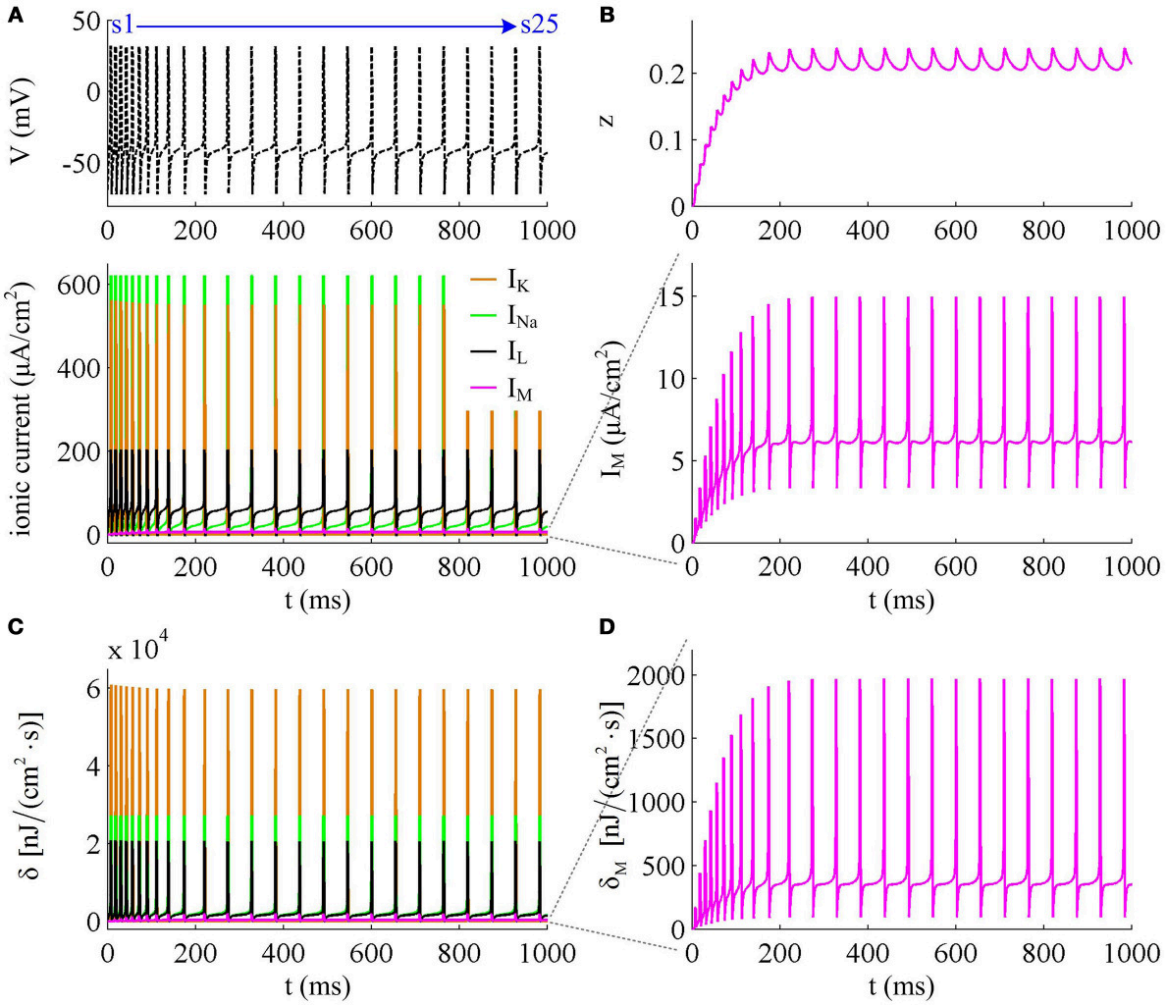
**Energy consumption rate involved in ionic currents with $I_{\text{S}}=43\mu \text{A}/\text{c}{\text{m}}^{2}$. (A)** *I*_Na_, *I*_K_, *I*_L_, and *I*_M_ underlying the spike trains triggered by $I_{\text{S}}=43\mu \text{A}/\text{c}{\text{m}}^{2}$. **(B)** *I*_M_ current and its activation variable *z*. **(C)** Energy consumption rate δ of each current. **(D)** Closer view of the energy consumption rate δ_M_ of *I*_M_ current.

**Figure 8:**
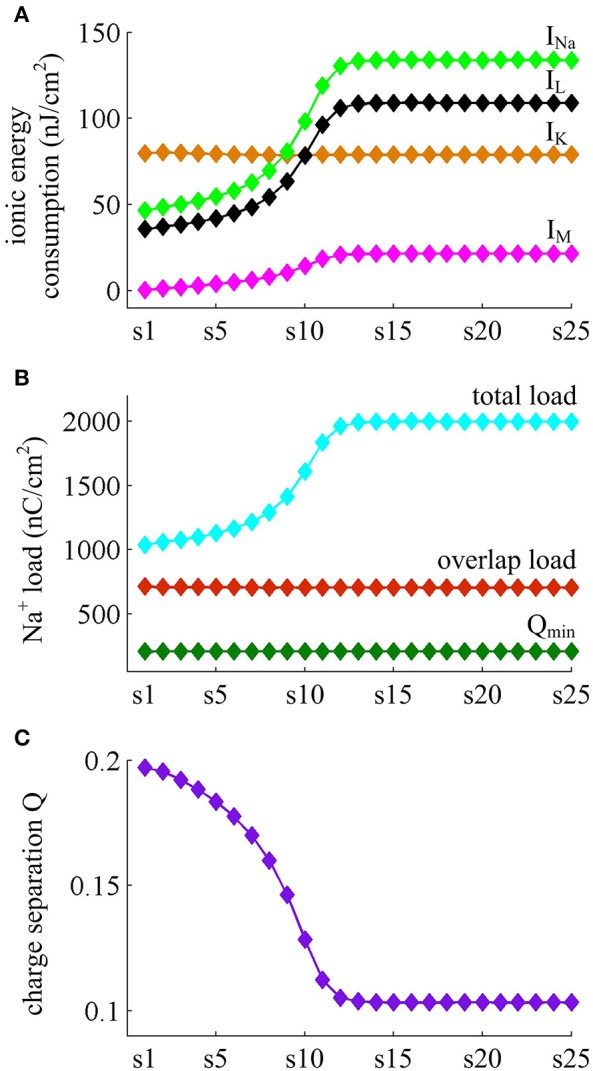
**Energy consumed by each channel and Na^+^ load with $I_{\text{S}}=43\mu \text{A}/\text{c}{\text{m}}^{2}$**. **(A)** Total energy cost by *I*_Na_, *I*_K_, *I*_L_, and *I*_M_ during each AP. **(B)** Total Na^+^ load, overlap Na^+^ load, and minimum Na^+^ charge Q_min_ during each AP. **(C)** Charge separation Q per spike during the course of SFA.

Figure 7 shows the ionic currents underlying the spike train elicited by $I_{\text{S}}=43\mu \text{A}/\text{c}{\text{m}}^{2}$. Their associated energy consumptions and Na^+^ load are summarized in Figure 8. At this value of *I*_S_, the adaptation variable *z* increases incrementally with each AP and decays between two APs (top panel, Figure 7B), which is unable to produce enough adaptation to terminate firing behavior. Then, the Prescott neuron continues to spike repetitively at a low rate in its steady state. In this case, the activation of *I*_M_ current reduces the rate of membrane depolarization preceding the spike initiation and extends relevant ISI. Such modulatory effects on subthreshold behavior result in the increase in total Na^+^ load (Figure 8B) as well as in its energy cost (Figure 8A) during the AP. They also increase the metabolic energy consumed by adaptation and leak channels (Figure 8A). However, the activation of *I*_M_ current hardly changes the repolarizing and hyperpolarizing dynamics of an AP (Figure 3D). Then, it results in few impacts on both the K^+^ energy consumption (Figure 8A) and the overlap Na^+^ load (Figure 8B). Further, the minimum Na^+^ charge Q_min_ required for the depolarization of the AP is unaffected by the activation of *I*_M_ (Figure 8B). But the presence of outward *I*_M_ in the rising phase of AP antagonizes inward Na^+^ and then effectively increases the Na^+^ load to achieve the depolarization. As a result, the charge separation Q of the AP decreases as *I*_M_ reduces firing rate (Figure 8C). That is, the Na^+^ influx is less efficiently used for AP depolarization with the activation of *I*_M_ current. Once adaptation variable *z* is sufficiently activated, the intensity of *I*_M_ stops increasing. It makes the firing behavior of the Prescott neuron reach steady state with a stable firing rate. Under this condition, the Na^+^ load per spike, the charge separation Q, and the associated metabolic energy involved in each ionic current all achieve their steady-state level. The results during the transient state in this case are similar to those obtained by $I_{\text{S}}=41\mu \text{A}/\text{c}{\text{m}}^{2}$.

### Effects of *I*_AHP_ on the energy cost of AP

**Figure 9:**
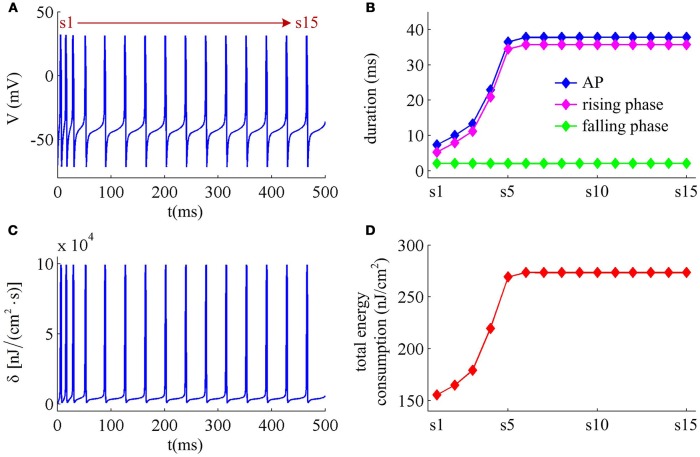
**Energy cost of spike trains generated in Prescott neuron with *I*_AHP_. (A)** Time courses of the spike train triggered by $I_{\text{S}}=47\mu \text{A}/\text{c}{\text{m}}^{2}$. There are 15 APs in total, and we number them from s1 to s15. **(B)** Duration of each AP, and its relevant rising and falling phase duration. **(C)** Energy consumption rate δ of the spike train. **(D)** Total energy cost per AP during the course of SFA.

**Figure 10:**
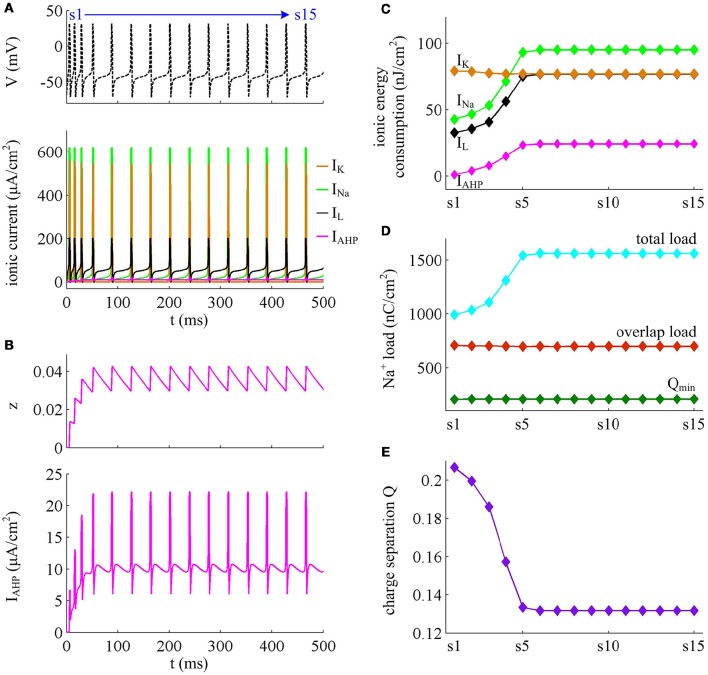
**Energy involved in the ionic currents in the case of *I*_AHP_. (A)** *I*_Na_, *I*_K_, *I*_L_, and *I*_AHP_ underlying the spike train triggered by $I_{\text{S}}=47\mu \text{A}/\text{c}{\text{m}}^{2}$ in Prescott model. **(B)** *I*_AHP_ current and its activation variable *z*. **(C)** Total energy consumption by each channel during an AP as the activation of *I*_AHP_ reduces firing rate. **(D)** Total Na^+^ load, overlap Na^+^ load, and minimum Na^+^ charge Q_min_ during each AP. **(E)** Charge separation Q per spike during the course of SFA.

With Prescott model, we have identified how the activation of *I*_M_ current modulates the energy cost of APs as it reduces firing rate. Here, we examine the effects of *I*_AHP_ current on the energy consumption of APs, which is summarized in Figures 9, 10. The relevant stimulus is $I_{\text{S}}=47\mu \text{A}/\text{c}{\text{m}}^{2}$. The inability of *I*_AHP_ to sustain activation at subthreshold potentials makes its adaptation variable *z* must increase with co-occurring spikes and exhibit invariable decrease between two APs (Figure 10B). Then, the *I*_AHP_ -mediated adaptation only reduces firing rate (Figure 9A), which is not sufficient to terminate repetitive spiking. By determining the energy cost of each AP in the simulated spike train, we find that the activation of *I*_AHP_ current results in the increase of total metabolic energy consumed by an AP during the course of SFA (Figure 9D).

From Figures 10A,B, one can observe that the intensity of *I*_AHP_ is much lower than the other three currents, which only reaches a peak of near 22μA/cm^2^. However, such weak current is able to participate in the process of spike initiation and reduce the rate of membrane depolarization prior to the AP, which further elongates the rising phase of AP and decreases firing rate (Figures 9A,B). These modulatory effects are similar to that induced by *I*_M_ -mediated adaptation. Then, as *I*_AHP_ activates, the Prescott neuron requires more inward Na^+^ load to depolarize its membrane (Figure 10D), and accordingly the energy cost by Na^+^ channel increases (Figure 10C). Once a spike is initiated, the *I*_AHP_ current has very few effects on neuronal dynamics, especially for the repolarizing component (Figure 9B). As a result, the activation of *I*_AHP_ current is unable to alter the energy consumption of outward K^+^ current (Figure 10C) or the overlap Na^+^ load during the repolarization period of AP (Figure 10D). By calculating the charge separation Q, we find that the minimum Na^+^ charge Q_min_ remains unchanged (Figure 10D) while the charge separation Q decays down (Figure 10E) as the activation of *I*_AHP_ current reduces firing rate. This means that the AP in Prescott neuron becomes less efficient to use Na^+^ influx during the course of SFA. All these impacts of *I*_AHP_ -mediated adaptation on the metabolic energy of APs during the transient state are in accordance with those induced by *I*_M_ -mediated adaptation.

### Effects of steady-state firing rate on the energy cost of AP

**Figure 11:**
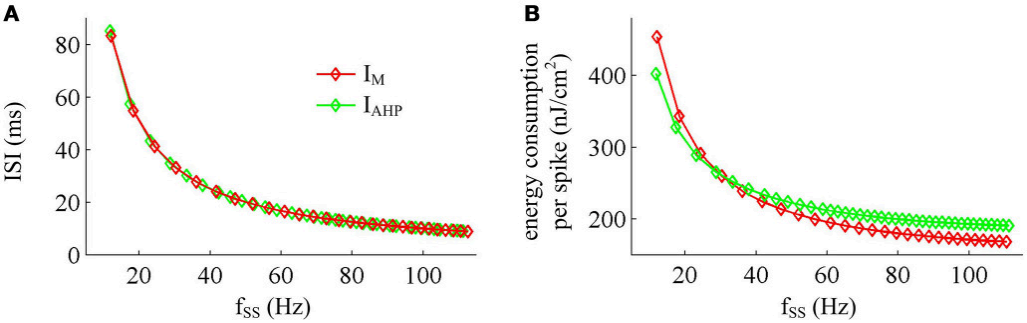
**Effects of *f*_SS_ on the energy cost of APs. (A)** Steady-state ISI as a function of *f*_SS_ in the case of *I*_M_ or *I*_AHP_ current. **(B)** Total energy cost per AP as a function of *f*_SS_.

**Figure 12:**
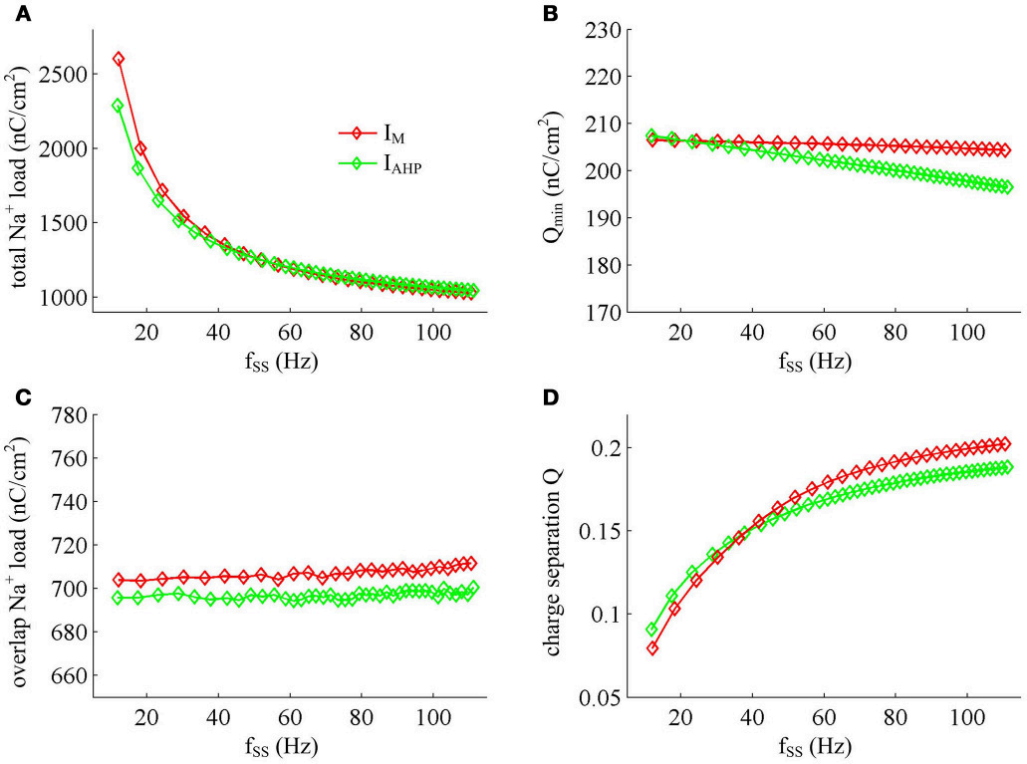
**Na^+^ load and charge separation Q as a function of *f*_SS_. (A)** Total Na^+^ load, **(B)** minimum Na^+^ charge Q_min_, **(C)** overlap Na^+^ load, and **(D)** charge separation Q during an AP in the observed range of *f*_SS_.

**Figure 13:**
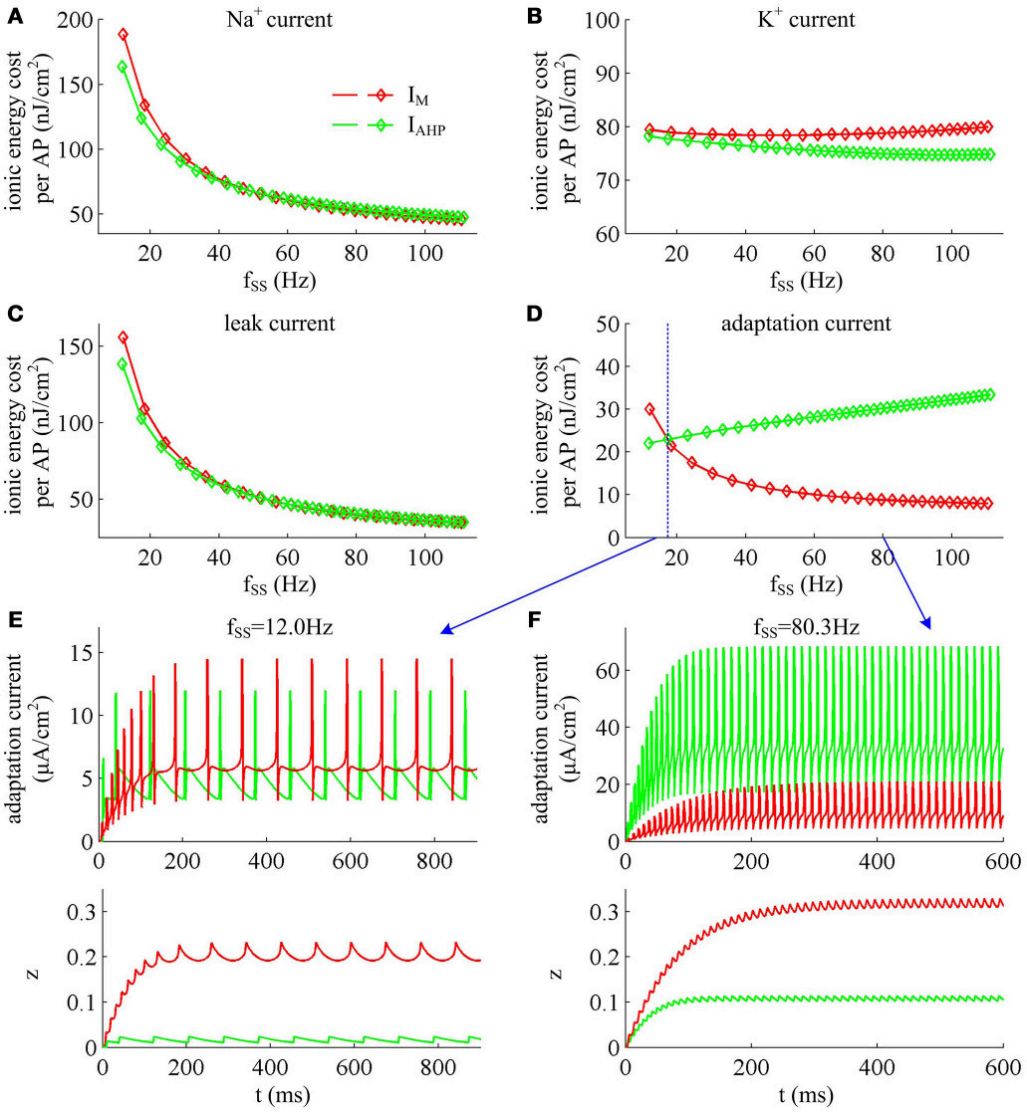
**Energy involved in ionic currents with different values of *f*_SS_**. Total metabolic energy consumed by **(A)** Na^+^, **(B)** K^+^, **(C)** leak, and **(D)** adaptation channels during an AP as *f*_SS_ is increased. **(E,F)** respectively show the time courses of two adaptation currents (i.e., *I*_M_ and *I*_AHP_) and their relevant activation variable *z* in the case of *f*_SS_ = 12.0Hz and 80.3Hz.

Previous sections have shown that the metabolic energy consumed by an AP increases as instantaneous firing rate decays down. Our next step is to use Prescott model to determine how the energy cost of APs with either *I*_M_ or *I*_AHP_ -mediated adaptation depends on steady-state firing rate *f*_SS_. In steady state, the adaptation currents have been sufficiently activated. As shown in Figure 11, one can find that the energy consumption in an AP varies inversely with *f*_SS_ for either *I*_M_ or *I*_AHP_ current. The *f*_SS_ in our study is calculated based on the reciprocal of steady-state ISI, and its value increase means the duration of relevant AP gets shorter (Figure 11A). Then, the total Na^+^ load in each AP decreases with *f*_SS_ for either form of adaptation current (Figure 12A), which leads to the reduction of energy consumption by Na^+^ or leak channels (Figures 13A,C). Further, as *f*_SS_ increases, there are no obvious variations in both the overlap Na^+^ load (Figure 12C) and the energy consumption in K^+^ channel (Figure 13B). These phenomena arise from the fact that the repolarization period of steady-state AP in the case of *I*_M_ or *I*_AHP_ both remains unchanged as *f*_SS_ varies. Unlike them, the charge separation Q is increased with *f*_SS_ (Figure 12D), which indicates that the Na^+^ entry is more efficiently used to generate the depolarization of steady-state AP in the case of high firing rate. This is due to that the minimum Na^+^ charge Q_min_ required for the depolarization of steady-state AP is less affected as *f*_SS_ is increased (Figure 12B).

Unlike Na^+^, K^+^ and leak currents, the energies consumed by *I*_M_ or *I*_AHP_ current show distinct evolutions in the observed range of *f*_SS_ (Figure 13D). To be specific, the energy consumption by *I*_AHP_ current during one AP increases with *f*_SS_, whereas the energy cost of *I*_M_ current varies inversely with *f*_SS_. At low firing rates, the metabolic energy consumed by *I*_M_ in an steady-state AP is larger than that of *I*_AHP_, whereas it becomes smaller than that of *I*_AHP_ at high firing rates. This arises from the different biophysical properties of two adaptation currents. With low firing rate, the intensity of either *I*_M_ or *I*_AHP_ is both very small. Due to the ability of activation prior to spike initiation, *I*_M_ increases from the subthreshold potentials, which is impossible for *I*_AHP_ current (Figure 13E). Then, the area under *I*_M_ current during an steady-state AP is larger than that of *I*_AHP_, which corresponds to higher energy cost. Increasing *f*_SS_ produces two disparate effects on the energy usage of adaptation currents. On one hand, the AP width is reduced as *f*_SS_ increases, which attenuates the energy consumption per spike for both *I*_M_ and *I*_AHP_. On the other hand, the activation level of adaptation variable *z* goes up as *f*_SS_ increases (Figures 13E,F), which makes *I*_M_ or *I*_AHP_ get stronger and then increases their energy consumption per spike. Since *I*_AHP_ has a relatively larger channel conductance (i.e., ${\bar{\text{g}}}_{\text{AHP}}=5\text{mS}/\text{c}{\text{m}}^{2}$), the increment of energy consumption induced by increasing its current intensity surpasses the decrement induced by reducing AP width. This enables the energy cost of *I*_AHP_ during an steady-state AP to increase with *f*_SS_. Compared with *I*_AHP_, the channel conductance of *I*_M_ is much lower, i.e., ${\bar{\text{g}}}_{\text{M}}=0.5\text{mS}/\text{c}{\text{m}}^{2}$. In this case, the increment of energy consumption is unable to surpass its decrement. Then, the energy cost of *I*_M_ during an AP varies inversely with *f*_SS_. As a result, the metabolic energy consumed by *I*_AHP_ becomes larger than that by *I*_M_ in the case of high firing rates.

In addition, the different biophysical properties of *I*_M_ and *I*_AHP_ currents are also translated into distinct modulations of the total energy cost per spike as *f*_SS_ increases. At small values of *f*_SS_, the steady-state AP in Prescott neuron with *I*_M_ current consumes higher energy than that with *I*_AHP_ current (Figure 11B), while its metabolic efficiency is lower (Figure 12D). At high firing rates, it becomes more efficient and consumes lower energy than that in the model with *I*_AHP_ current.

### Effects of SFA on AP-related energy in detailed hodgkin-huxley type model

The above findings are obtained in Prescott neuron, which reproduces SFA by incorporating either adaptation current in a two-dimensional ML type model. However, the Prescott neuron neglects some details of the realistic model, such as Na^+^ inactivation. During the process of AP initiation, the inactivation of Na^+^ current reduces the availability of Na^+^ channels that can be used to depolarize membrane and thus directly affects the excitability of the cell. In particular, it has been shown that the Na^+^ inactivation plays a considerable role in both the overlap Na^+^ load and the energy efficiency of AP (Crotty and Levy, 2007; Sengupta et al., 2010; Kandel et al., 2012). In this section, we investigate how the activation of *I*_M_ or *I*_AHP_ affects the AP-related energy with a detailed conductance-based model involving Na^+^ inactivation. The model is a Hodgkin-Huxley (HH) type model proposed by Ermentrout (1998). See supplementary material for the detailed parameters and expressions of each ionic channel in the model.

**Figure 14:**
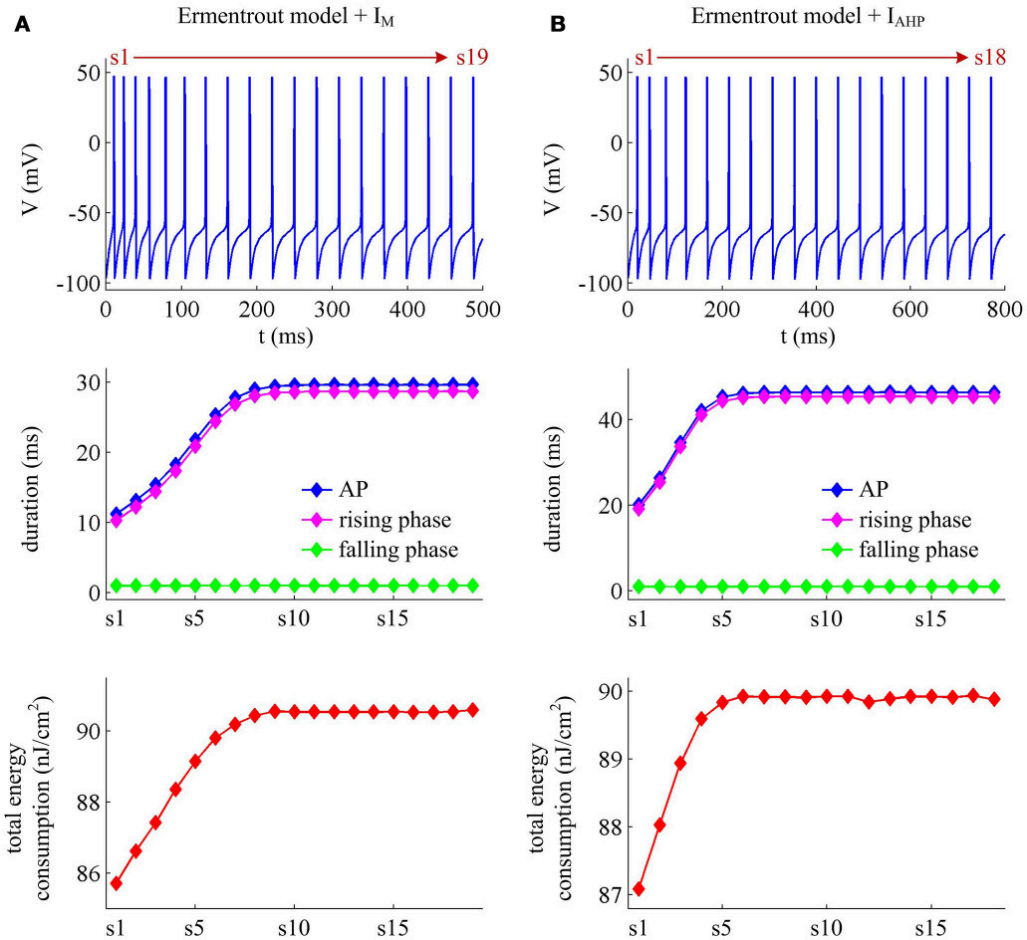
**Energy cost of spike trains generated in Ermentrout neuron. (A,B)** respectively show the spike trains and relevant energy cost per AP simulated in the model with *I*_M_ or *I*_AHP_. The value of *I*_S_ is 3μA/cm^2^ for *I*_M_ current and 1.2μA/cm^2^ for *I*_AHP_ current. Note that the values of *I*_S_ here are much lower than those for Prescott neuron. This is determined by the conductance-based models being considered. The biophysical properties of ionic currents make the level of excitability in Ermentrout neuron higher than that in Prescott neuron. Then, the former requires much lower stimulus to generate APs than the latter. For the detailed calculation of energy cost in Ermentrout neuron, please see Supplementary material.

**Figure 15:**
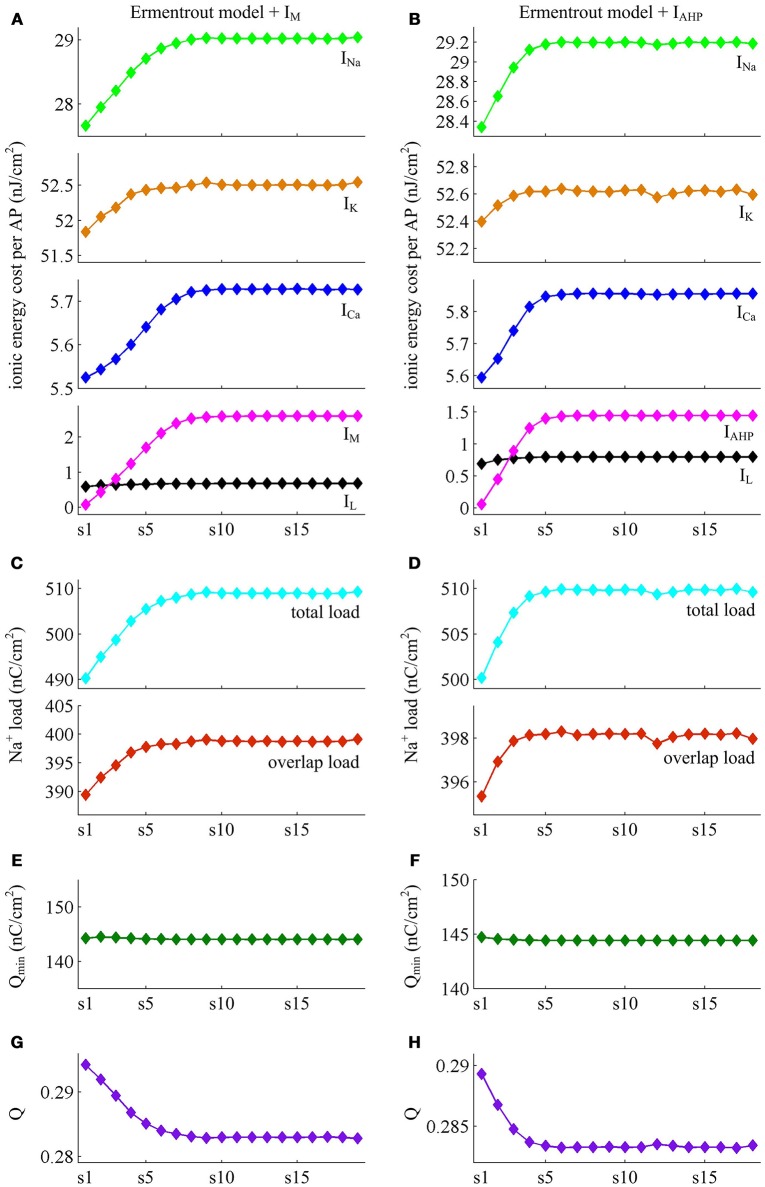
**Energy involved in the ionic currents of Ermentrout neuron. (A,B)** summarize the energy cost by each ionic channel during an AP with *I*_M_ ($I_{\text{S}}=3\mu \text{A}/\text{c}{\text{m}}^{2}$) or *I*_AHP_ ($I_{\text{S}}=1.2\mu \text{A}/\text{c}{\text{m}}^{2}$) current. The total Na^+^ load and overlap Na^+^ load per spike in two cases are respectively shown in **(C,D)**. **(E,F)** depict the minimum Na^+^ charge Q_min_ needed for the depolarization of relevant AP. **(G,H)** show the charge separation Q per spike during the course of SFA.

We apply constant current to stimulate Ermentrout neuron to generate APs. Then, we compute the metabolic energy consumed in each AP and in its underlying ionic currents. The Na^+^ load and charger separation Q of relevant APs is also calculated for either spike train. Similar to Prescott neuron, the activation of *I*_M_ or *I*_AHP_ in Ermentrout neuron extends the duration of the rising phase of AP while does not alter the falling phase as it reduces firing rate. Therefore, the AP-related energy increases during the course of SFA (Figure 14). However, there is small increase in the energy cost of K^+^ current from one AP to the next as *I*_M_ or *I*_AHP_ activates (Figures 15A,B). Meanwhile, the overlap Na^+^ load per AP also shows a slight increase during the course of SFA (bottom panels, Figures 15C,D). These differences from Prescott neuron may arise from the present of Na^+^ inactivation in Ermentrout neuron. For leak channel, its energy cost per spike remains almost unchanged as the activation of either adaptation current reduces firing rate (Figures 15A,B). This is due to that the channel conductance of passive *I*_L_ (i.e., ${\text{g}}_{\text{L}}=0.1\text{mS}/\text{c}{\text{m}}^{2}$) here is much smaller than active currents. Further, the minimum Na^+^ charge Q_min_ needed for AP depolarization remains nearly unchanged with the activation of *I*_M_ or *I*_AHP_ (Figures 15E,F), and therefore the charge separation Q is reduced from one AP to the next (Figures 15G,H). This indicates that the Ermentrout neuron becomes less efficiently to use Na^+^ entry for the depolarization of AP during the course of SFA.

Overall the impacts of *I*_M_ or *I*_AHP_ current on the charge separation Q per spike, the total Na^+^ load per spike, the minimum Na^+^ charge Q_min_ as well as the energy cost during an AP are all in accordance with those from simple Prescott model. Therefore, the predictions of the AP-related energy and AP efficiency during the course of SFA are reproducible in detailed HH type model, which are unaffected by the presence of Na^+^ inactivation.

## Discussion and conclusions

We have used single-compartmental conductance-based models to investigate the relationships between the APs during the course of SFA, the currents that generate them and the energies they consume. The SFA is generated by incorporating an adaptation current, i.e., *I*_M_ or *I*_AHP_, in the model. Both of *I*_M_ and *I*_AHP_ are inhibitory K^+^ currents, which cause SFA by introducing a form of slow negative feedback to the excitability of the cell. The energy consumption related to SFA is calculated using the novel approach proposed by Moujahid et al. (2011). This method allows us to precisely measure the energy cost involved in each AP as well as in its underlying ionic currents during the course of SFA.

Our simulations show that the activation of *I*_M_ or *I*_AHP_ both causes the increase in the energy cost of AP as it reduces firing rate. In fact, the intensity of either adaptation current during an AP is much weaker than other currents, especially Na^+^ and K^+^. However, they are able to participate in the process of AP initiation, and their activation directly slows down the rate of membrane depolarization preceding the spike. As a consequence, the width of depolarizing component of the AP extends and the firing rate is reduced, i.e., SFA occurs. It is known that the influx of Na^+^ ions is responsible for membrane depolarization, then the Na^+^ load increases with the activation of *I*_M_ or *I*_AHP_. Since Na^+^ influx drives Na^+^/K^+^ ATPase activity (Alle et al., 2009; Harris et al., 2012; Kandel et al., 2012), the associated energy cost in Na^+^ channel increases from one AP to the next. The leak current is passive and varies with membrane voltage, and thus widening AP also leads to the increase of energy cost in this channel. The activation of *I*_M_ or *I*_AHP_ has few effects on the falling phase of AP. That is because here two outward adaptation currents are very weak and the outward *I*_K_ dominates the repolarization and hyperpolarization of AP. In this case, the energy cost in K^+^ channel is less affected by the activation of either adaptation current. These modulations result in that the total metabolic energy increases from one AP to the next as *I*_M_ or *I*_AHP_ activates.

With conductance-based models, we find that the AP-related energy varies inversely with the instantaneous firing rate during the transient response to current steps. In fact, the inverse relationship between them has already been observed in the cells from neocortex, hippocampus, thalamus, and squid giant axon (Moujahid and d'Anjou, 2012; Sengupta et al., 2013; Moujahid et al., 2014). This indicates that the AP-related metabolic energy is tightly related to its period, since it directly determines the amount of ions involved in the process of AP generation. It is worth noting that the firing rate in earlier modeling studies (Moujahid and d'Anjou, 2012; Sengupta et al., 2013; Moujahid et al., 2014) is increased by stronger input current, higher temperature, larger channel density, or bigger cell diameter. Unlike them, the instantaneous firing rate in present study is reduced by the activation of slow adaptation current. This is an intrinsic biophysical factor of the cell, which is controlled by the gating variable of inhibitory K^+^ currents on slower timescales than fast dynamics of AP generation.

There is temporal overlap between inward Na^+^ and outward K^+^ currents in the falling phase of AP, which has been shown to be the major determinant of AP efficiency (Sengupta et al., 2010; Howarth et al., 2012; Moujahid and d'Anjou, 2012). As *I*_M_ or *I*_AHP_ activates, the overlap between two ions in either conductance-based model varies little from one AP to the next. This is due to that their activation does not alter the repolarization component of relevant AP. Meanwhile, the activation of either *I*_M_ or *I*_AHP_ also does not alter the minimum Na^+^ charge that is required for the depolarization of AP. To measure the energy efficiency of AP as firing rate decays down, we calculate the charge separation as the ratio of minimum Na^+^ charge to total Na^+^ charge per AP (Alle et al., 2009; Moujahid and d'Anjou, 2012; Moujahid et al., 2014). This is a dimensionless measure, which evaluates the proportion of Na^+^ entry that is confined to the rising phase of the AP. Our simulations show that the charge separation Q per spike is reduced as either adaptation current activates. This indicates that the influx of Na^+^ is less efficiently used to generate the depolarization of AP during the course of SFA. In fact, the activation of *I*_M_ or *I*_AHP_ in the rising phase of AP results in the overlaps between with the Na^+^ influx current, which increases the Na^+^ charge influx required for depolarization. Such overlap is in effect similar to the overlap between the Na^+^ influx and the repolarizing K^+^ current, which effectively increases the Na^+^ load to achieve depolarization. As a result, the activation of either adaptation current makes the AP less efficient as it reduces firing rate. This finding suggests that slow adaptation currents are the potential biophysical causes for regulating the energy efficiency of neural computation. It also highlights that an AP with higher energy cost corresponds to a lower metabolic efficiency (Alle et al., 2009; Howarth et al., 2012; Moujahid and d'Anjou, 2012; Sengupta et al., 2013; Moujahid et al., 2014).

There have been several studies on the energy efficiency of neuronal information processing. Moujahid and d'Anjou (2012) find that increasing temperature results in the decrease in both energy cost per spike and overlap Na^+^ load (i.e., increased efficiency). Sengupta et al. (2010) report that the reductions in Na^+^ or K^+^ conductance have a limited ability to improve AP efficiency, while reducing the time constant for Na^+^ inactivation is more effective. Recently, Sengupta et al. (2013) show that increasing cell size increases energy cost per spike while reduce energy efficiency. In our earlier study (Yi et al., 2015a), we have shown that depolarizing AP threshold improves energy efficiency by reducing overlap Na^+^ load. In present study, we observe that the activation of *I*_M_ or *I*_AHP_ increases AP-related energy and reduces AP efficiency while has few effects on the overlap Na^+^ load. Our stimulations also reveal that the APs in Ermentrout neuron are more efficient than those in Prescott neuron. These studies indicate that there are a variety of potential causes for differential energy efficiency. Any changes in the biophysics or structures of the neurons could potentially lead to the changes in their energy usage. This gives the diversity of metabolic efficiency in different cell types. Further, the findings also highlight previous proposal that there is no direct relationship between AP shape and its energy efficiency (Sengupta et al., 2010).

It is known that the energy supply of brain determines its information processing power (Attwell and Gibb, 2005). In fact, much of brain's energies are used to reverse the ion fluxes that generate APs and synaptic currents. For example, Howarth et al. (2012) recently report that the energy “budget” for neural computation in the cerebral cortex includes synaptic processes (59%), APs (21%), and resting potentials (20%). In the cerebellar cortex, the majority of signaling energy use is on the maintenance of resting potentials (54%) and postsynaptic receptors (22%), while APs only account for 17%. However, the energy supply available for the brain is limited, which brings serious metabolic constraints on brain networks, CNS neurons as well as their functions (Attwell and Gibb, 2005; Howarth et al., 2010, 2012; Lewis et al., 2014; Ju et al., 2016). This implies that there will be metabolically efficient strategies for neural coding. Our simulations reveal that the SFA caused by slow inhibitory K^+^ currents makes an AP less efficiently use Na^+^ influx for its depolarization as firing rate is reduced. This seems like that the intrinsically generated SFA is not a potential factor that contributes to efficient coding. But plenty of studies have shown that SFA, as a common property of CNS neurons, effectively enhances the encoding ability of a neuron to incoming signals on multiple timescales (Wang, 1998; Liu and Wang, 2001; Benda and Herz, 2003; Prescott et al., 2006; Wark et al., 2007; Prescott and Sejnowski, 2008; Benda et al., 2010; Ladenbauer et al., 2014). In this sense, there may be some internal connections between the ionic mechanisms of SFA and the metabolic efficiency of neural coding. Therefore, it requires further theoretical or experimental studies to determine their relationship.

A number of literatures have used ion counting approach to estimate the energy costs associated with neural computation in the cerebral cortex (Attwell and Laughlin, 2001), the hippocampal and thalamus (Sengupta et al., 2010), the neocortex and cerebellum (Howarth et al., 2010, 2012), and the olfactory bulb (Nawroth et al., 2007). This approach is performed by calculating the minimum Na^+^ entry used to generate the membrane voltage change during the AP. The measured Na^+^ influx is converted into ATP consumption by using the fact that the Na^+^/K^+^-ATPase consumes one ATP when it extrudes three Na^+^ ions out of the cell. Since there is temporal overlap between Na^+^ and K^+^ currents during an AP, the energy value obtained by ion counting should be corrected by applying a multiplication factor. For squid giant axon, a factor of 4 is used (Hodgkin, 1975). For cerebral cortex cells, the factor is 1.24 (Carter and Bean, 2009). For cerebellar cells, the factor ranges from 1.04 for granule cells (Sengupta et al., 2010) to 2 for Purkinje cells (Carter and Bean, 2009). Such temporal overlap leads to the controversy about the value of factor used to estimate the energy use. Unlike ion counting, the calculation of energy consumption in present study is based on the biophysical nature and the circuit characteristic of neuron models. It enables us to find an analytical expression of the metabolic energy involved in the dynamics of the model. This method does not include any prior hypothesis about the stoichiometry of ions or the extent of the overlap. Therefore, it can address the above problem of ion counting. When estimating the energy costs of other cell types, it only requires developing the relevant model while does not have to determine the value of multiplication factor.

The SFA plays a crucial role in the information processing of a neuron and thus affects its function. In particular, the different activation properties of *I*_M_ and *I*_AHP_ have been shown to produce distinct effects on how a neuron encodes synaptic inputs, such as frequency-current curves, gain of spiking, ISI variability, or spike-timing reliability (Prescott et al., 2006; Prescott and Sejnowski, 2008; Benda et al., 2010; Ladenbauer et al., 2014; Yi et al., 2015b). Our simulations (Figures 11–13) demonstrate that the differences in the biophysics of *I*_M_ and *I*_AHP_ also result in distinct energy usages for generating APs, which is comparable to these earlier predictions. In current study, we have not formally investigated the energetics involved in neural coding related to SFA. However, the basic principles obtained by our simulations could provide deep and new insights into how two ionic mechanisms of SFA participate in the energy use on the information processing in neurons, which are useful to interpret their functional significance in neural coding. Even so, all our findings need to be verified with experimental approaches in future studies.

There are other mechanisms may induce SFA at the single cell level except *I*_M_ and *I*_AHP_. One is the sodium-activated K^+^ currents (*I*_KNa_), which is a slow current instantaneously gated by the intracellular concentration of Na^+^ (Wang et al., 2003). It results in SFA on much longer timescales than *I*_M_ or *I*_AHP_, which usually lasts about many seconds. The slow inactivation of Na^+^ current is also a potential mechanism for inducing SFA (Fleidervish et al., 1996; Benda et al., 2010), which is a third gating variable for inward Na^+^ current. Note that this dynamics is on a timescale of about a second, which is much slower than the Na^+^ inactivation involved in the Ermentrout model. It reduces firing rate by slowly reducing the availability of Na^+^ channels for depolarizing membrane. Further, a dynamic threshold that is incremented by each spike event may also cause a model neuron to reproduce SFA (Bibikov and Ivanitskíí, 1985; Liu and Wang, 2001; Benda et al., 2010). We have previously characterized the AP-related energy for different dynamics of spike threshold (Yi et al., 2015a). But the threshold dynamics in that study is associated with the fast initiating process of single AP, which occurs on a very short timescale. The dynamic threshold for generating SFA is on a slower timescale ranging from about tens of ms to several seconds. In future research, it requires to incorporate these potential mechanisms in neuron models to separately investigate how they participate in the AP-related energy as they induce SFA.

To conclude, our study has obtained a better interpretation of the basic principles about how two adaptation mechanisms affect the energy cost of APs as they reduce firing rate to constant stimulus. Through characterizing the metabolic energy involved in the ionic currents underlying APs, we have provided a biophysical link between SFA and AP-related energy at the single cell level. The findings here are comparable to earlier modeling and experimental predictions about the energy cost of APs. As the computational unit of the CNS, the energy supply available determines the information processing power of neurons. A substantial portion of total energy budget for a neuron is used to generate and propagate sequences of APs. Therefore, identifying the AP-related energy involved in the firing patterns associated with SFA is essential and necessary for deeply understanding how various subcellular processes underlying neural information processing work.

## Author contributions

Conceived and designed the work: GY, JW, HL, XW, and BD, Performed the simulations: GY, JW, and XW, Analyzed and interpreted the data: GY, JW, and BD. Wrote the paper: GY, JW, HL, and XW.

### Conflict of interest statement

The authors declare that the research was conducted in the absence of any commercial or financial relationships that could be construed as a potential conflict of interest. The reviewer RR and handling Editor declared their shared affiliation, and the handling Editor states that the process nevertheless met the standards of a fair and objective review.
