# Supplementary material for: Metabolic Energy of Action Potentials Modulated by Spike Frequency Adaptation
Source: Front Neurosci. 2016 Nov 17;10:534. doi: 10.3389/fnins.2016.00534 (PMC5112251; doi:10.3389/fnins.2016.00534)
Supplement: Supplementary file 1 [file DataSheet1.docx]

Supplementary Material

Metabolic energy of action potentials modulated by spike frequency adaptation

Guo-Sheng Yi, Jiang Wang, Hui-Yan Li, Xi-Le Wei, Bin Deng*

*** Correspondence:** Corresponding Author: [dengbin@tju.edu.cn](mailto:dengbin@tju.edu.cn)

# Ermentrout neuron model

This model is a HH type model, which includes five ionic currents (Ermentrout, 1998). They are fast Na^+^ current $I_{\text{Na}}$ with activation *m* and inactivation *h*, delayed rectifier K^+^ current $I_{\text{K}}$ with activation *n*, voltage-gated Ca^2+^ current $I_{\text{Ca}}$, slow adaptation current $I_{\text{adapt}}$, and leak current $I_{\text{L}}$. The model is described by

$\text{C}\frac{\text{d}V}{\text{d}t}=I_{\text{S}}-I_{\text{Na}}-I_{\text{K}}-I_{\text{Ca}}-I_{\text{adapt}}-I_{\text{L}}$ (S1)

The ionic currents are

(S2)

Here $w_{\infty}(V)=1/\left\{1+\exp\left[-(V+25)/5\right]\right\}$. Relevant parameters are $\text{C}=1\text{μF}/\text{cm}^{2}$, $\text{E}_{\text{Na}}=50\text{mV}$, $\text{E}_{\text{K}}=-100\text{mV}$, $\text{E}_{\text{Ca}}\text{=120mV}$, $\text{E}_{\text{L}}=-67\text{mV}$, $\overline{\text{g}}_{\text{Na}}=100\text{mS}/\text{cm}^{2}$, $\overline{\text{g}}_{\text{K}}=80\text{mS}/\text{cm}^{2}$, $\overline{\text{g}}_{\text{Ca}}\text{ }=1\text{mS}/\text{cm}^{2}$, $\text{g}_{\text{L}}=0.1\text{mS}/\text{cm}^{2}$. The gating variables in above obey the following differential equation

$\frac{\text{d}y}{\text{d}t}=\alpha_{y}(V)(1-y)-\beta_{y}(V)(1-y),\text{ }y=m,\text{ }n,\text{ }h$ (S3)

where

$\begin{matrix} \alpha_{m}(V)=0.32(54+V)/\left\{1-\exp\left[-(V+54)/4\right]\right\} \\ \beta_{m}(V)=0.28(V+27)/\left\{\exp\left[(V+27)/5\right]-1\right\} \\ \alpha_{h}(V)=0.128\exp\left[-(50+V)/18\right] \\ \beta_{h}(V)=4/\left\{1+\exp\left[-(V+27)/5\right]\right\} \\ \alpha_{n}(V)=0.032(V+52)/\left\{1-\exp\left[-(V+52)/5\right]\right\} \\ \beta_{n}(V)=0.5\exp\left[-(57+V)/40\right] \end{matrix}$ (S4)

The adaptation current $I_{\text{adapt}}$ is either $I_{\text{M}}$ or $I_{\text{AHP}}$. For $I_{\text{M}}$-mediated adaptation, there is

$\begin{matrix} I_{\text{M}}=\overline{\text{g}}_{\text{M}}z(V-\text{E}_{\text{K}}) \\ \frac{\text{d}z}{\text{d}t}=0.01\left[\alpha_{z}(V)-z\right] \\ \alpha_{z}(V)=1/\left\{1+\exp\left[-(V+20)/5\right]\right\} \end{matrix}$ (S5)

where $\text{ }\overline{\text{g}}_{\text{M}}=5\text{mS}/\text{cm}^{2}$ and $\text{ }\overline{\text{g}}_{\text{AHP}}=0\text{mS}/\text{cm}^{2}$. For $I_{\text{AHP}}$-mediated adaptation, there is

(S6)

Here $\text{ }\overline{\text{g}}_{\text{AHP}}\text{ }=5\text{mS}/\text{cm}^{2}$, and $\text{ }\overline{\text{g}}_{\text{M}}=0\text{mS}/\text{cm}^{2}$.

According to Equation (9)-(12), the energy consumption rate in Ermentrout model can be written as

$\delta=I_{\text{S}}V-\delta_{\text{Na}}-\delta_{\text{K}}-\delta_{\text{Ca}}-\delta_{\text{adapt}}-\delta_{\text{L}}$ (S7)

where

(S8)
